# Habitual Iron Supplementation Associated with Elevated Risk of Chronic Kidney Disease in Individuals with Antihypertensive Medication

**DOI:** 10.3390/nu16142355

**Published:** 2024-07-20

**Authors:** Xiaoyan Ma, Jiali Lv, Shuai Zhang, Xiaofeng Zhang, Xia Lin, Shengxu Li, Lin Yang, Fuzhong Xue, Fan Yi, Tao Zhang

**Affiliations:** 1 Department of Biostatistics, School of Public Health, Cheeloo College of Medicine, Shandong University, Jinan 250012, China; 202216373@mail.sdu.edu.cn (X.M.); jialilv@mail.sdu.edu.cn (J.L.); zhangshuai522@mail.sdu.edu.cn (S.Z.); 202216394@mail.sdu.edu.cn (X.Z.); 202236450@mail.sdu.edu.cn (X.L.); xuefzh@sdu.edu.cn (F.X.); 2 Institute for Medical Dataology, Cheeloo College of Medicine, Shandong University, Jinan 250012, China; 3 Children’s Minnesota Research Institute, Children’s Minnesota, Minneapolis, MN 55404, USA; lishx@hotmail.com; 4 Department of Cancer Epidemiology and Prevention Research, Cancer Research & Analytics, Cancer Care Alberta, Alberta Health Services, Calgary, AB T2V 0N5, Canada; lin.yang@albertahealthservices.ca; 5 Departments of Oncology and Community Health Sciences, Cumming School of Medicine, University of Calgary, Calgary, AB T2V 0N5, Canada; 6 The Key Laboratory of Infection and Immunity of Shandong Province, Department of Pharmacology, School of Basic Medical Sciences, Shandong University, Jinan 250012, China

**Keywords:** chronic kidney disease, iron supplementation, hypertension, antihypertension medication

## Abstract

The aim of this study was to examine the effects of habitual iron supplementation on the risk of CKD in individuals with different hypertensive statuses and antihypertension treatment statuses. We included a total of 427,939 participants in the UK Biobank study, who were free of CKD and with complete data on blood pressure at baseline. Cox proportional hazards regression models were used to examine the adjusted hazard ratios of habitual iron supplementation for CKD risk. After multivariable adjustment, habitual iron supplementation was found to be associated with a significantly higher risk of incident CKD in hypertensive participants (HR 1.12, 95% CI 1.02 to 1.22), particularly in those using antihypertensive medication (HR 1.21, 95% CI 1.08 to 1.35). In contrast, there was no significant association either in normotensive participants (HR 1.06, 95% CI 0.94 to 1.20) or in hypertensive participants without antihypertensive medication (HR 1.02, 95% CI 0.90 to 1.17). Consistently, significant multiplicative and additive interactions were observed between habitual iron supplementation and antihypertensive medication on the risk of incident CKD (*p* all interaction < 0.05). In conclusion, habitual iron supplementation was related to a higher risk of incident CKD among hypertensive patients, the association might be driven by the use of antihypertensive medication.

## 1. Introduction

Chronic kidney disease (CKD) is a growing global public health challenge [1], with hypertension emerging as a predominant factor in both high-income and middle-income countries [2,3]. Although the effect of impaired kidney function on iron homeostasis has been established [4,5], the direct impact of iron deficiency or overload on the development and progression of kidney disease is less explored and remains uncertain. Iron is an essential mineral for the maintenance of functional homeostasis in the human body [6,7,8], and the iron supplement is frequently used to prevent iron deficiency anemia [9,10]. Despite the increasing use of mineral supplements, including iron supplements, in many European countries [11], the benefits of dietary mineral supplements for non-communicable diseases lack robust evidence [8,10,12]. Recent studies suggest that dietary or supplemental iron overload may increase the risk of multiple health outcomes, including CKD and other chronic diseases [13,14,15,16,17,18]. Moreover, animal experiments suggest that iron overload significantly contributes to CKD, possibly due to lipid peroxidation and pro-oxidative stress caused by iron supplements [19,20,21,22]. However, there is a lack of large-scale epidemiologic investigation on the association between habitual iron supplementation and CKD risk.

Hypertension is characterized by an upregulation of the angiotensin–aldosterone system (RAAS) [23], with a high prevalence (>30%) in the global adult population [24,25]. The use of iron supplements would further increase renal injury due to oxidative stress and hypoxia from iron overload [26,27] Cross-sectional studies have shown that elevated ferritin concentrations, an indicator of iron overload, were positively associated with hypertension risk [28], suggesting potential interactions between iron levels and hypertension. Moreover, there are concerns about the potential nephrotoxic effects of iron supplements, especially when taken in high dosages or in combination with conventional medications [29,30]. Identifying the link between habitual iron supplementation and CKD risk is of public health importance. Further, it remains unclear whether heterogeneity in such a link exists in the connections between iron supplements and CKD incidence among individuals with different hypertensive or antihypertensive statuses.

To address these gaps in knowledge, we aimed to elucidate the connections between habitual iron supplementation and CKD incidence among individuals with varying hypertensive or antihypertensive statuses and explore whether the hypertensive or antihypertensive statuses would modify the relationship between habitual iron supplementation and CKD.

## 2. Materials and Methods

### 2.1. Study Cohort

The UK Biobank is a large prospective cohort study of 500,000 participants aged 40–69 years throughout the United Kingdom during 2006–2010. The study protocol has been detailed in prior publications [31,32,33]. Participants contributed their health data through touchscreen questionnaires and underwent physical measurements. Upon enrollment, blood, urine, and saliva samples were systematically gathered for subsequent genotyping and biomarker analyses. The ethical approval of the UK Biobank was obtained from the North West Multi-Centre Research Ethics Committee (London, UK), and all participants provided written informed consent.

**Figure 1:**
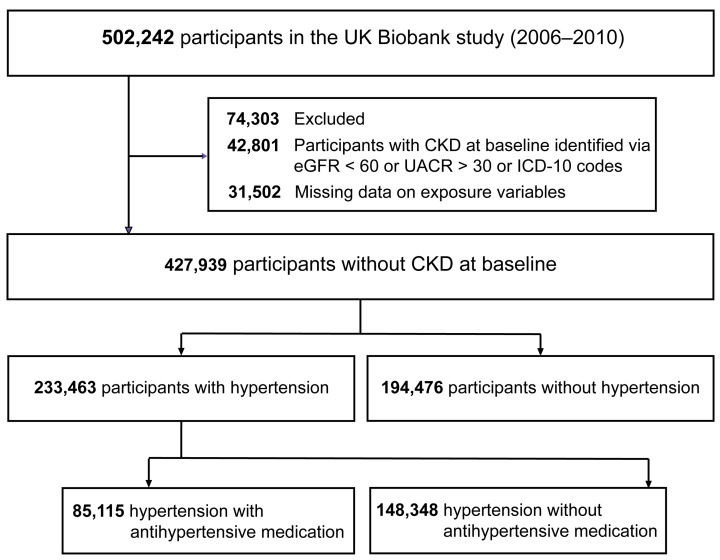
Flowchart of the participant inclusion in this study. Abbreviations: CKD = chronic kidney disease; eGFR = estimated glomerular filtration rate; UACR = urinary albumin–creatinine ratio; ICD-10 = the international statistical classification of diseases and related health problems 10th revision.

In the current study, we excluded the prevalent cases of CKD at baseline (*n* = 42,801), individuals lacking data on systolic blood pressure (SBP) or diastolic blood pressure (DBP) (*n* = 26,611), and those with incomplete information on the use of iron supplements (*n* = 4891) (Figure 1). At last, a total of 427,939 participants were analyzed in the present study.

### 2.2. Ascertainment of Hypertension and Antihypertensive Medication Use

Individuals classified with hypertension met at least one of the following criteria: (1) self-reported physician-diagnosed cases; (2) current use of antihypertensive medication; or (3) SBP ≥ 140 mmHg or DBP ≥ 90 mmHg [34]. After excluding missing data on antihypertensive medication, hypertension was further divided into two categories based on medication use: hypertension with antihypertensive medication and hypertension without antihypertensive medication. Information on antihypertensive medication was obtained through the question “Do you regularly take any of the following medications?”. Men and women could choose multiple answers from a medication list that included blood pressure medication. Antihypertensive medication was scored as “0 = no” and “1 = yes”.

### 2.3. Assessment of Iron Supplementation

Based on the electronic questionnaire at baseline, the habitual iron supplementation of participants was assessed by asking the question “Do you regularly take any of the following?”, with a response list of supplements, including iron. Consistent with prior research [35,36], we categorized habitual iron supplementation as “0 = no” and “1 = yes”. 

### 2.4. Assessment of Chronic Kidney Disease

The definition of incident CKD was based on the International Statistical Classification of Diseases, 10th Revision (ICD-10) codes (N03, N06, N08, N11, N12, N13, N14, N15, N16, N18, N19, N20, and N21) in any primary care data, hospital inpatient data, and death register records. The date and cause of hospital admissions were obtained via linkage with the Hospital Episode Statistics for England, Scottish Morbidity Records for Scotland, and the Patient Episode Database for Wales. 

Prevalent cases of CKD were defined as an estimated glomerular filtration rate (eGFR) < 60 mL/min per 1.73 m^2^ or a urinary albumin–creatinine ratio (UACR) > 30 mg/g at baseline [2,24,37] or identified through the listed ICD-10 codes recorded at recruitment. The eGFR was estimated using the Chronic Kidney Disease Epidemiology Collaboration (CKD-EPI) [38,39].

### 2.5. Assessment of Covariates

We used information from the touchscreen questionnaire at recruitment to assess several potential confounders: sociodemographic characteristics (age, sex, Townsend deprivation index [TDI], and ethnicity), lifestyle factors (smoking status, alcohol drinking status, other mineral supplementation, vitamin supplementation, physical activity, and healthy diet), body mass index (BMI), aspirin use, comorbidities (anemia, diabetes, and hypercholesterolemia).

Information on sociodemographic characteristics was collected from the central registry at recruitment and updated by the participants. According to the American Heart Association recommendations on physical activity for health [40], we categorized participants into three groups based on the time spent in moderate and vigorous activity in minutes each week: inactive, insufficient, and active. A healthy diet was based on the consumption of at least 4 of 7 commonly eaten food groups [41]. BMI was calculated as the weight (kg) divided by height squared (m^2^). Additional details regarding covariates can be found in the Methods section provided in the Supplementary Materials [42,43].

### 2.6. Statistical Analyses

The statistical description of baseline characteristics is presented as mean (standard deviation) for continuous variables and the number (percentage) for categorical variables. The *t* tests or χ^2^ tests were used to compare the difference between participants who used iron supplements and those who did not in both hypertensives and normotensives at baseline. Missing data of the covariates were replaced with median values for continuous variables and marked as “missing” for categorical variables.

Multivariable Cox proportional hazards regression models were used to calculate the adjusted hazard ratio (HR) and 95% confidence intervals (95% CI) for the association between habitual iron supplementation and risk of CKD in individuals with different hypertensive or antihypertensive statuses. The Schoenfeld residuals method was used to test the proportional hazard assumption of the Cox models [44], and no violation of this assumption was seen in our analyses. Three sets of models were used to adjust potential confounders. In model 1, we adjusted for baseline age (years), sex (male or female), race (White, Asian, Black, mix, or others), and the TDI (continuous). Model 2 further adjusted for alcohol drinking status (never drinking, former drinking, or current drinking), smoking status (never smoker, former smoker, or current smoker), vitamin supplementation (yes or no), other mineral supplementation (yes or no), physical activity (inactive, insufficient, or active), healthy diet (yes or no), BMI (continuous), and aspirin use (yes or no). In model 3, additional adjustments were made for the presence of anemia (yes or no), diabetes (yes or no), and hypercholesterolemia (yes or no).

To examine how habitual iron supplementation affects CKD risk in individuals with different hypertensive and antihypertensive statuses, we tested the multiplicative and additive interactions. The multiplicative interactions were tested using the likelihood ratio test by including an additional product term in the models. According to Rothman [45], the interactions on an additive scale were calculated using relative risks, including the relative excess risk due to interaction (RERI), the attributable proportion (AP), and the synergy index (SI). An absence of additive interaction would be indicated if the 95% CI for RERI and AP contains 0 and for SI includes 1.

Participants were divided into four groups based on hypertensive status (hypertension or normotension) and habitual iron supplementation status (using or not) to investigate the joint associations. After adjusting for covariates in model 3, HRs for CKD risk in different groups were calculated, with normotension without iron supplements as the reference. Similarly, hypertensive individuals with different antihypertensive statuses (with or without antihypertensive medication) were classified into four groups for comparison with normotension without iron supplements.

Association analyses were further stratified by the following group: age (≥60 or <60 years), sex (male or female), BMI (≥30 or <30 kg/m^2^), smoking status (current/former or never), alcohol drinking status (current or former/never), anemia (yes or no), diet habit (healthy diet or unhealthy diet), physical activity (active or inactive/insufficient), vitamin supplements use (yes or no), diabetes (yes or no), hypercholesterolemia (yes or no), and aspirin use (yes or no) in individuals with different hypertensive and antihypertensive status.

To test the robustness of our findings, several sensitivity analyses were conducted. First, we conducted a 1:4 propensity score-matched cohort to repeat the main analyses. We used a generalized linear model to calculate the propensity scores after adjusting the covariates in model 3. Second, we investigated the association between habitual iron supplementation and chronic renal failure (CRF) risk in participants with different hypertensive and antihypertensive statuses. The ICD-10 code for CFR is N18, which represents the most common composition of CKD. Third, we repeated the analyses among individuals without missing covariates. Fourth, normotension was redefined according to the European Renal Association and International Society of Hypertension guidelines (SBP < 130 mmHg and DBP < 85 mmHg) [34], and additional analyses were conducted on individuals with newly classified hypertensive status.

All statistical analyses were conducted using R software version 4.3.1 (R Foundation), and a two-sided *p* value < 0.05 was considered as statistical significance.

## 3. Results

### 3.1. Baseline Characteristics

A total of 427,939 participants (mean age 56.4 [SD 8.1] years; 233,633 [54.6%] females; and 403,435 [94.3%] White individuals) were included in this study (Figure 1), with a median follow-up duration of 14.8 years, of which 233,463 (54.6%) had hypertension. Among these hypertensive individuals, 85,115 (36.5% of 233,463) reported using antihypertensive medication at baseline. Table 1 summarizes the baseline characteristics of the study participants by hypertensive status (hypertension or normotension) and iron supplementation status (users or non-users). Overall, 14,082 (3.3%) participants reported habitual iron supplementation at baseline, higher among those with normotension than those who had hypertension (4.0% vs. 2.7%, *p* < 0.001). In respect to hypertensive status, iron supplement users were generally younger, more likely to be female, non-smokers, non-current alcohol drinkers, and inclined to use minerals, vitamins, and other dietary supplements; maintained a healthy diet; had a higher prevalence of anemia; and tended to take more aspirin but exhibited a lower prevalence of hypercholesterolemia. Additionally, among individuals with hypertension, iron supplement users had a higher prevalence of diabetes compared to non-users, whereas this trend was not observed among normotensive individuals.

Conversely, hypertensive individuals with medication, compared to those without, were older and more likely to be male, non-smokers, and non-alcohol drinkers, with a higher prevalence of anemia, hypercholesterolemia, diabetes, and aspirin use (Supplementary Table S1). The distribution of SBP and DBP in participants with different hypertensive and antihypertensive statuses indicated lower values in those using antihypertensive medication, presumably attributable to the medication’s action (Supplementary Figures S1 and S2).

Over a median follow-up of 14.8 years, 6.5% of the study participants (*n* = 27,829) reported incident CKD. Supplementary Figure S3 shows the cumulative incidence of CKD in different populations, highlighting differences in CKD incidence between iron supplement users and non-users in subpopulations. Particularly, in the hypertensive group using antihypertensive medication, the incidence of CKD was significantly higher among iron supplement users compared to non-users.

### 3.2. Habitual Iron Supplementation and CKD

Table 2 presents the associations between habitual iron supplementation and risk of CKD. After adjusting for demographic characteristics (model 1), habitual iron supplementation significantly increased the risk of CKD in individuals with hypertension (HR 1.14, 95% CI 1.05 to 1.24, *p* = 0.003). This association persisted even after adjusting for lifestyle factors (model 2) (HR 1.17, 95% CI 1.07 to 1.27, *p* < 0.001) or comorbidities (model 3) (HR 1.12, 95% CI 1.02 to 1.22, *p* = 0.013). However, no significant association was found between habitual iron supplementation and CKD incidence in normotensive individuals (*p* > 0.05). Notably, the risk of habitual iron supplementation for CKD incidence was particularly pronounced in hypertensive individuals with antihypertensive medication, with HRs (95% CI) of 1.29 (1.15 to 1.44, *p* < 0.001) in model 1, 1.30 (1.16 to 1.45, *p* < 0.001) in model 2, and 1.21 (1.08 to 1.35, *p* < 0.001) in model 3, whereas no significant association was observed in those without antihypertensive medication (*p* > 0.05).

Table 3 shows the evident multiplicative and additive interactions between habitual iron supplementation and antihypertensive medication use on the risk of CKD after adjusting for covariates in model 3 (*p*_all interaction_ < 0.05). Based on measures of additive interactions (RERI, AP, and SI), there was a 0.25 relative excess risk of CKD due to the additive interaction between habitual iron supplementation and antihypertensive medication. The proportion attributable to additive interactions was 17.0%. In addition, the risk of CKD was 2.33 times higher in individuals exposed to both risk factors compared to those exposed to a single risk factor. On the other hand, the interaction between habitual iron supplementation and hypertensive status was not significant on either multiplicative or additive scales (Supplementary Table S3).

**Figure 2:**
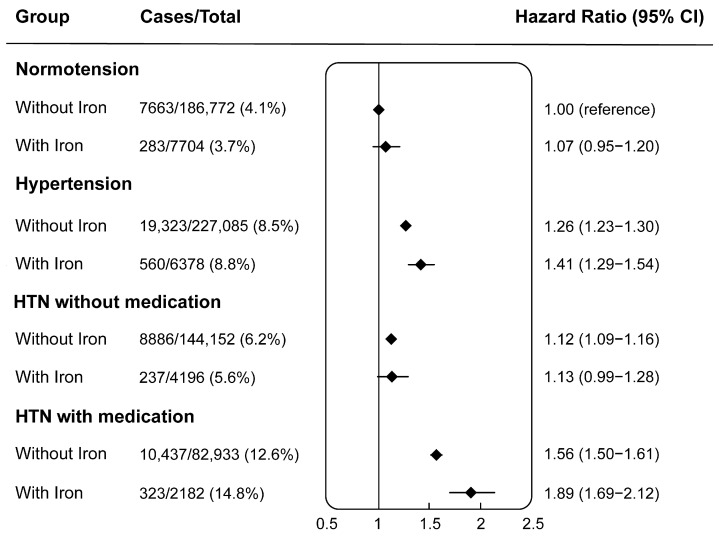
Joint associations (HR, 95% CI) of habitual iron supplement use and hypertensive status (normotension or hypertension) as well as antihypertensive status of participants with hypertension (without medication or with medication) with chronic kidney disease incidence. Notes: Without iron indicates the participants not using iron supplements. With iron indicates the participants using iron supplements. Without medication indicates participants with hypertension not using antihypertensive medication. With medication indicates participants with hypertension using antihypertensive medication. HRs (95% CI) were adjusted for age (continuous), sex (male or female), race (White, Asian, Black, mix, or others), the Townsend deprivation index (continuous), alcohol drinking status (never drinking, former drinking, or current drinking), smoking status (never smoker, former smoker, or current smoker), vitamin supplementation (yes or no), other mineral supplementation (yes or no), physical activity (inactive, insufficient, or active), healthy diet (yes or no), BMI (continuous), anemia (yes or no), diabetes (yes or no), hypercholesterolemia (yes or no), and aspirin use (yes or no). Healthy diet was defined as at least 4 of the following 7 food groups: fruits ≥ 3 servings/day; vegetables ≥ 3 servings/day; fish ≥ 2 servings/day; processed meats ≤ 1 serving/week; unprocessed red meats ≤ 1.5 servings/week; whole grains ≥ 3 servings/day; and refined grains ≤ 1.5 servings/day.

Figure 2 presents the combined effect of habitual iron supplementation and hypertensive/antihypertensive status in participants, with normotensive individuals without iron supplement use as the reference. After adjusting for covariates in model 3, hypertensive individuals with habitual iron supplementation exhibited a notably higher risk of CKD incidence (HR 1.41, 95% CI 1.29 to 1.54). Furthermore, habitual iron supplementation increased the risk of CKD incidence in hypertensive individuals with antihypertensive medication, as indicated by HRs [95% CI] of 1.89 [1.69 to 2.12] versus 1.56 [1.50 to 1.61] compared to the reference.

### 3.3. Subgroup and Sensitivity Analyses

We conducted subgroup analyses and consistently observed results when stratifying analyses by age, sex, BMI, smoking status, alcohol drinking status, anemia, diet habits, physical activity, vitamin supplement use, diabetes, hypercholesterolemia, and aspirin use (Supplementary Table S2). The trend of these associations between habitual iron supplementation and risk of CKD was stronger in individuals with a higher BMI (≥30 kg/m^2^) or an unhealthy diet. Those associations also demonstrated some population heterogeneity among certain stratification factors, such as age, sex, BMI, smoking status, alcohol drinking status, vitamin supplements use, diabetes status, hypercholesterolemia status, and aspirin use.

The associations and interactions in study population subgroups remained robust across separate sensitivity analyses: (1) in the 1:4 propensity score-matched cohort (Supplementary Table S4); (2) if the outcome was limited to CRF (Supplementary Tables S5 and S6); (3) if individuals who had missing data on covariates in model 3 were excluded (Supplementary Tables S7 and S8); and (4) if normotension was defined as individuals with SBP < 130 mmHg and DBP < 85 mmHg (Supplementary Tables S9 and S10). In line with the phenomenon shown in Figure 2, the combined effect of habitual iron supplementation and hypertensive/antihypertensive status remained consistent across all sensitivity analyses (Supplementary Figures S4–S7).

## 4. Discussion

In this large-scale prospective study, we observed a 12% higher CKD incidence among hypertensive individuals with habitual iron supplementation, which was not observed in the normotensive group. Furthermore, our analysis revealed that elevated CKD risk associated with iron supplementation was particularly pronounced for hypertensive individuals who reported antihypertensive medication use. Importantly, these associations remained independent of traditional risk factors, including age, sex, race, TDI, alcohol drinking status, smoking status, use of other supplements, physical activity, healthy diet, BMI, aspirin use, anemia, diabetes, and hypercholesterolemia. The robustness of these associations was confirmed through various stratified and sensitivity analyses. Notably, our study also uncovered the interaction between iron supplementation and antihypertensive medication, suggesting a combined effect that goes beyond the individual impacts of these factors. These findings emphasize the complexity of the relationship among iron supplementation, hypertension, antihypertensive medication, and incident CKD risk. 

Our findings on the relationship between iron supplements and CKD in hypertension and antihypertensive medication subgroups align with the conclusions of previous studies. For instance, numerous previous animal studies and reviews propose that exceeding recommended levels of iron supplements may lead to various adverse health impacts [13,46,47]. A national cohort study conducted in the United States revealed a significant increase in the risk of incident CKD associated with oral iron replacement [17]. A recent review also has concluded that there is limited evidence supporting the preventive role of iron supplements [12]. Additionally, the absence of a significant association between iron supplements and CKD in normotensive individuals is also consistent with prior studies that found no significant impact of iron supplements on the overall health status of the general population [48].

However, the existing literature lacks clarity regarding the diverse impacts of these associations within different population subgroups. While hypertension is a well-established risk factor for CKD [24,49], limited research has explored whether habitual iron supplementation amplifies the risk of CKD in individuals with hypertension, especially in those taking antihypertensive medication. To the best of our knowledge, this study revealed for the first time potential population heterogeneity in the relationship between iron supplements and CKD in a large prospective cohort.

A potential explanation for the heterogeneity of the relationship between iron supplements and CKD across different subgroups is that hypertensive individuals, particularly those using antihypertensive medication, have increased lipid peroxidation and oxidative metabolism [26,50,51,52]. Excessive use of iron supplements may oxidize lipids and increase oxidative stress, and the excess iron via the Fenton reaction may induce lipid peroxidation, which leads to ferroptosis [20,21,27,53,54]. Ferroptosis is dependent on iron and lipid metabolism and contributes to vascular calcification in CKD [55,56,57]. Conversely, in normotensive individuals, the absence of an association between iron supplementation and CKD incidence could be attributed to the lack of hypertensive characteristics. Additionally, people on antihypertensive medication tend to have a higher prevalence of comorbidities linked to CKD incidence, such as diabetes and hypercholesterolemia.

On the other hand, the significant interaction implies that the combined effect of iron supplements and antihypertensive medication was greater than the sum of their individual substances. Several potential mechanisms may explain this interaction. Iron overload induced by iron supplements might lead to the formation of stable iron-drug chelates with conventional medications, potentially altering drug action and metabolism [58,59]. Moreover, the iron overload-induced oxidative stress contradicts the antioxidant effects of some antihypertensive drugs and significantly impacts their efficacy [60]. Furthermore, animal experiments have confirmed that certain antihypertensive medications can disturb iron homeostasis as evidenced by a slight increase in kidney iron levels [29], which interact with iron supplements and result in kidney damage and an increased CKD risk. 

The higher risk of CKD with iron supplementation raises doubts about the appropriateness of taking iron supplements among individuals using medications for treating hypertension. Future studies are needed to replicate the present findings and to elucidate underlying mechanisms for the increased risk of CKD associated with iron supplementation in patients with hypertension. Before a definite answer regarding iron supplementation can be reached, caution is needed in recommending iron supplements in patients with hypertension.

Our study has several major strengths. Firstly, it clarifies the potential adverse effects of iron supplements on CKD incidence in an observational study, employing a large-scale prospective cohort study design. Secondly, the inclusion of nearly half a million participants ensures a substantial number of outcome events, providing ample statistical power for comprehensive analyses across individuals with varying hypertensive or antihypertensive statuses. Thirdly, the availability of detailed information on socioeconomic characteristics, lifestyle, and other covariables facilitates adjustment for potential confounders. Lastly, comprehensive subgroup and sensitivity analyses were conducted to demonstrate the robustness of those associations.

Several limitations should also be considered. Firstly, the UK Biobank contains only categorical data on the use of iron supplements, without detailed information on dosage, formulation, and duration of use. This precludes further evaluation of the dose–response relationship and the effects of different iron supplement formulations and supplementation durations. Secondly, exposure information was only measured at baseline. Repeated measurements of iron supplementation are needed to determine the correlation between changes in iron supplementation patterns and the risk of CKD incidence. Thirdly, specific information on medication types was not collected, making it difficult to assess potential differences in the interaction between various types of antihypertensive medications and iron supplements. Fourthly, residual or unknown confounding bias cannot be completely excluded due to the limitation of the observational study design. Fifthly, participants recruited in this study were mostly middle-aged or older white adults. It might limit the generalizability to other racial or age groups. Finally, we should acknowledge that the sparse data might inflate the hazard ratio and odds ratio in the SI of additive interactions [61]. Moreover, according to Susan F. Assmann [62], the SI was statistically unstable, so the RERI and AP, which were used to determine the robustness of additive interactions, are more reliable.

## 5. Conclusions

In conclusion, this large-scale prospective study highlights a significant link between habitual iron supplementation and an increased risk of incident CKD in hypertensive patients, particularly in those taking antihypertensive medication. These findings suggest that individuals with hypertension, especially those using antihypertensive medication, should exercise caution when considering long-term iron supplement use. To comprehensively understand the benefits and potential adverse effects of iron supplements, especially in populations with hypertension, additional clinical and experimental investigations are warranted.

## Figures and Tables

**Table 1:** Baseline characteristics of the study participants.

Variable	Total	Hypertension			Normotension		
		Without Iron	With Iron	*p*-Value	Without Iron	With Iron	*p*-Value
Participants, *n*	427,939	227,085	6378		186,772	7704	
CKD, *n* (%)	27,829 (6.5)	19,323 (8.5)	560 (8.8)	0.458	7663 (4.1)	283 (3.7)	0.066
Antihypertensive medication, *n* (%)	85,115 (19.9)	82,933 (36.5)	2182 (34.2)	<0.001	-	-	-
Follow time, years	14.4 (2.1)	14.3 (2.4)	14.2 (2.5)	<0.001	14.6 (1.8)	14.5 (1.7)	0.049
Age, years	56.4 (8.1)	58.6 (7.4)	56.7 (8.0)	<0.001	53.8 (8)	51.4 (7.7)	<0.001
Sex female, *n* (%)	233,633 (54.6)	109,124 (48.1)	3914 (61.4)	<0.001	114,644 (61.4)	5951 (77.2)	<0.001
TDI	−1.3 (3.1)	−1.4 (3.1)	−0.5 (3.4)	<0.001	−1.4 (3)	−0.7 (3.3)	<0.001
Ethnic White, *n* (%)	403,435 (94.3)	215,559 (94.9)	5452 (85.5)	<0.001	175,726 (94.1)	6698 (86.9)	<0.001
Smoking status, *n* (%)				<0.001			<0.001
Never	234,646 (54.8)	119,498 (52.6)	3627 (56.9)		106,890 (57.2)	4631 (60.1)	
Former	147,027 (34.4)	84,976 (37.4)	2126 (33.3)		57,674 (30.9)	2251 (29.2)	
Current	44,269 (10.3)	21,510 (9.5)	590 (9.3)		21,367 (11.4)	802 (10.4)	
Alcohol drinking status, *n* (%)				<0.001			<0.001
Never	18,205 (4.3)	9498 (4.2)	471 (7.4)		7692 (4.1)	544 (7.1)	
Former	14,772 (3.5)	7800 (3.4)	375 (5.9)		6217 (3.3)	380 (4.9)	
Current	394,034 (92.1)	209,369 (92.2)	5514 (86.5)		172,387 (92.3)	6764 (87.8)	
Mineral supplements, *n* (%)	45,417 (10.6)	20,403 (9.0)	2801 (43.9)	<0.001	18,883 (10.1)	3330 (43.2)	<0.001
Vitamin supplements, *n* (%)	137,788 (32.2)	67,452 (29.7)	5123 (80.3)	<0.001	58,966 (31.6)	6247 (81.1)	<0.001
Physical activity *, *n* (%)				<0.001			<0.001
Inactive	43,859 (10.2)	24,542 (10.8)	637 (10.0)		18,061 (9.7)	619 (8.0)	
Insufficient	66,371 (15.5)	36,473 (16.1)	941 (14.8)		27,964 (15.0)	993 (12.9)	
Active	308,366 (72.1)	160,306 (70.6)	4590 (72.0)		137,523 (73.6)	5947 (77.2)	
Healthy diet ^†^, *n* (%)	256,559 (60.0)	132,419 (58.3)	4104 (64.3)	<0.001	114,755 (61.4)	5281 (68.5)	
Fruits	212,643 (50.4)	111,578 (49.9)	3672 (58.7)	<0.001	92,997 (50.4)	4396 (57.8)	<0.001
Vegetables	347,302 (82.8)	184,282 (83.0)	5220 (84.0)	0.036	151,443 (82.5)	6357 (83.8)	0.005
Fish	221,083 (52.2)	121,769 (54.2)	3490 (55.4)	0.062	91,954 (49.7)	3870 (50.7)	0.098
Processed meats	294,441 (69.0)	151,372 (66.9)	4584 (72.2)	<0.001	132,517 (71.2)	5968 (77.7)	<0.001
Unprocessed meats	211,649 (50.1)	105,999 (47.3)	3427 (54.8)	<0.001	97,504 (52.8)	4719 (61.9)	<0.001
Whole grains	43,737 (10.3)	24,142 (10.7)	564 (8.9)	<0.001	18,397 (9.9)	634 (8.3)	<0.001
Refine grains	328,315 (77.1)	170,451 (75.5)	4934 (78.0)	<0.001	146,617 (78.8)	6313 (82.3)	<0.001
BMI, kg/m^2^	27.3 (4.7)	28.4 (4.9)	28.4 (5.4)	0.859	26 (4.1)	25.5 (4.2)	<0.001
SBP, mmHg	137.2 (18.1)	148.4 (15.5)	146.9 (15.6)	<0.001	123.8 (9.8)	121.4 (10.3)	<0.001
DBP, mmHg	82 (9.9)	86.9 (9.2)	86.9 (9.4)	0.719	76.2 (7)	75.2 (7.2)	<0.001
Anemia, *n* (%)	24,201 (5.7)	11,092 (4.9)	751 (11.8)	<0.001	11,420 (6.1)	938 (12.2)	<0.001
HC, *n* (%)	69,836 (16.3)	55,506 (24.4)	1322 (20.7)	<0.001	12,646 (6.8)	362 (4.7)	<0.001
Diabetes, *n* (%)	21,824 (5.1)	17,155 (7.6)	571 (9.0)	<0.001	3938 (2.1)	160 (2.1)	0.006
Aspirin use, *n* (%)	56,582 (13.2)	42,039 (18.5)	1219 (19.1)	0.230	12,738 (6.8)	586 (7.6)	0.008

Notes: With iron indicates the participants using iron supplements. Without iron indicates the participants not using iron supplements. Data are present as mean ± SD for continuous variables and *n* (%) for categorical variables. Abbreviations: CKD = chronic kidney disease; TDI = Townsend deprivation index; BMI = body mass index; SBP = systolic blood pressure; DBP = diastolic blood pressure; HC = hypercholesterolemia. *, Physical activity was defined as inactive (no documented moderate or vigorous physical activity), insufficient (moderate activity < 150 min/week and vigorous activity < 75 min/week), and active (moderate activity < 150 min/week and/or vigorous activity < 75 min/week). ^†^, Healthy diet was defined as at least 4 of the following 7 food groups: fruits ≥ 3 servings/day; vegetables ≥ 3 servings/day; fish ≥ 2 servings/day; processed meats ≤ 1 serving/week; unprocessed red meats ≤ 1.5 servings/week; whole grains ≥ 3 servings/day; and refined grains ≤ 1.5 servings/day.

**Table 2:** Associations (HR, 95% CI) of habitual iron supplementation with risk of chronic kidney disease in participants with different hypertensive statuses (normotension or hypertension) and antihypertensive statuses (without medication or with medication).

Groups	Without Iron (Case/Total)	With Iron (Case/Total)	Model 1 *		Model 2 ^†^		Model 3 ^‡^	
			HR (95% CI)	*p*-Value	HR (95% CI)	*p*-Value	HR (95% CI)	*p*-Value
Normotension	7663/186,772 (4.1%)	283/7704 (3.7%)	1.03 (0.92–1.17)	0.579	1.08 (0.95–1.22)	0.243	1.06 (0.94–1.20)	0.319
Hypertension	19,323/227,085 (8.5%)	560/6378 (8.8%)	1.14 (1.05–1.24)	0.003	1.17 (1.07–1.27)	<0.001	1.12 (1.02–1.22)	0.013
Without medication	8886/144,152 (6.2%)	237/4196 (5.6%)	1.01 (0.89–1.15)	0.880	1.04 (0.91–1.18)	0.590	1.02 (0.90–1.17)	0.741
With medication	10,437/82,933 (12.6%)	323/2182 (14.8%)	1.29 (1.15–1.44)	<0.001	1.30 (1.16–1.45)	<0.001	1.21 (1.08–1.35)	0.001

Notes: Without iron indicates the participants not using iron supplements. With iron indicates the participants using iron supplements. Without medication indicates participants with hypertension not using antihypertensive medication. With medication indicates participants with hypertension using antihypertensive medication. Model 1 *: adjusted for age (continuous), sex (male or female), race (White, Asian, Black, mix, or others), and the Townsend deprivation index (continuous). Model 2 ^†^: further adjusted for alcohol drinking status (never drinking, former drinking, or current drinking), smoking status (never smoker, former smoker, or current smoker), vitamin supplementation (yes or no), other mineral supplementation (yes or no), physical activity (inactive, insufficient, or active), healthy diet (yes or no), body mass index (continuous), and aspirin use (yes or no). Model 3 ^‡^: further adjusted for anemia (yes or no), diabetes (yes or no), and hypercholesterolemia (yes or no). Healthy diet was defined as at least 4 of the following 7 food groups: fruits ≥ 3 servings/day; vegetables ≥ 3 servings/day; fish ≥ 2 servings/day; processed meats ≤ 1 serving/week; unprocessed red meats ≤ 1.5 servings/week; whole grains ≥ 3 servings/day; and refined grains ≤ 1.5 servings/day.

**Table 3:** Multiplicative and additive interactions between habitual iron supplementation and antihypertensive status (without medication or with medication) on chronic kidney disease risk in participants with hypertension.

	Model 1 *	Model 2 ^†^	Model 3 ^‡^
**Multiplicative interaction**			
HR (95% CI)	1.30 (1.10–1.54)	1.26 (1.07–1.50)	1.21 (1.02–1.43)
*p*-value	0.002	0.007	0.030
**Additive interaction**			
RERI (95% CI)	0.41 (0.24–0.58)	0.33 (0.18–0.49)	0.25 (0.09–0.41)
*p*-value	<0.001	<0.001	0.001
AP (95% CI)	0.23 (0.12–0.34)	0.21 (0.09–0.33)	0.17 (0.04–0.30)
*p*-value	<0.001	<0.001	0.004
SI (95% CI)	2.14 (1.06–4.28)	2.40 (0.85–6.73)	2.33 (0.63–8.65)
*p*-value	<0.001	<0.001	<0.001

Abbreviations: RERI = the relative excess risk due to interaction; AP = the proportion attributable to interaction; SI = the synergy index. Model 1 *: adjusted for age (continuous), sex (male or female), race (White, Asian, Black, mix, or others), and the Townsend deprivation index (continuous). Model 2 ^†^: further adjusted for alcohol drinking status (never drinking, former drinking, or current drinking), smoking status (never smoker, former smoker, or current smoker), vitamin supplementation (yes or no), other mineral supplementation (yes or no), physical activity (inactive, insufficient, or active), healthy diet (yes or no), body mass index (continuous), and aspirin use (yes or no). Model 3 ^‡^: further adjusted for anemia (yes or no), diabetes (yes or no), and hypercholesterolemia (yes or no). Healthy diet was defined as at least 4 of the following 7 food groups: fruits ≥ 3 servings/day; vegetables ≥ 3 servings/day; fish ≥ 2 servings/day; processed meats ≤ 1 serving/week; unprocessed red meats ≤ 1.5 servings/week; whole grains ≥ 3 servings/day; and refined grains ≤ 1.5 servings/day.

## Data Availability

All data used in this study are publicly accessible from UK Biobank via their standard data access procedure at https://www.ukbiobank.ac.uk (accessed on 8 February 2024).

## Supplementary Materials

The following supporting information can be downloaded at: https://www.mdpi.com/article/10.3390/nu16142355/s1, Table S1. Baseline characteristics of participants with hypertension in different antihypertensive status; Table S2. Stratified analysis of the associations (HR, 95% CI) of habitual iron supplementation with risk of chronic kidney disease in participants with different hypertensive statuses (normotension or hypertension) and antihypertensive statuses (without medication or with medication); Table S3. Multiplicative and additive interactions between habitual iron supplementation and hypertensive status (normotension or hypertension) on chronic kidney disease risk; Table S4. Associations (HR, 95% CI) of habitual iron supplementation with risk of chronic kidney disease in participants with different hypertensive statuses (normotension or hypertension) and antihypertensive statuses (without medication or with medication) in the 1:4 propensity score-matched cohort; Table S5. Associations (HR, 95% CI) of habitual iron supplementation with risk of chronic renal failure (ICD-10 code: N18) in participants with different hypertensive statuses (normotension or hypertension) and antihypertensive statuses (without medication or with medication) (case = 17,575; Table S6. Multiplicative and additive interactions between habitual iron supplementation and antihypertensive status (without medication or with medication) on chronic renal failure (ICD-10 code: N18) in participants with hypertension; Table S7. Associations (HR, 95% CI) of habitual iron supplementation with risk of chronic kidney disease in participants with different hypertensive statuses (normotension or hypertension) and antihypertensive statuses (without medication or with medication) after excluding people with missing covariates; Table S8. Multiplicative and additive interactions between habitual iron supplementation and antihypertensive status (without medication or with medication) on chronic kidney disease in participants with hypertension after excluding people with missing covariates; Table S9. Associations (HR, 95% CI) of habitual iron supplementation with risk of chronic kidney disease in participants with different hypertensive statuses (normotension or hypertension) and antihypertensive statuses (without medication or with medication) when the normotension was defined as individuals with normal BP (systolic BP < 130 mmHg and diastolic BP < 85 mmHg); Table S10. Multiplicative and additive interactions between habitual iron supplementation and antihypertensive status (without medication or with medication) on chronic kidney disease in participants with hypertension when the normotension was defined as individuals with normal BP (systolic BP < 130 mmHg and diastolic BP < 85 mmHg); Figure S1. The distribution of systolic blood pressure in participants with different hypertensive statuses (normotension or hypertension) and antihypertensive statuses (without medication or with medication); Figure S2. The distribution of diastolic blood pressure in participants with different hypertensive statuses (normotension or hypertension) and antihypertensive statuses (without medication or with medication); Figure S3. Cumulative CKD events rate varies among participants with different hypertensive statuses (normotension or hypertension) and antihypertensive statuses (without medication or with medication); Figure S4. Joint associations (HR, 95% CI) of habitual iron supplement use and hypertensive status (normotension or hypertension) as well as antihypertensive status of participants with hypertension (without medication or with medication) with chronic kidney disease incidence in the 1:4 propensity score-matched cohort; Figure S5. Joint associations (HR, 95% CI) of habitual iron supplement use and hypertensive status (normotension or hypertension) as well as antihypertensive status of participants with hypertension (without medication or with medication) with chronic renal failure (ICD-10 code: N18) incidence; Figure S6. Joint associations (HR, 95% CI) of habitual iron supplement use and hypertensive status (normotension or hypertension) as well as antihypertensive status of participants with hypertension (without medication or with medication) with chronic kidney disease incidence after excluding people with missing covariates; Figure S7. Joint associations (HR, 95% CI) of habitual iron supplement use and hypertensive status (normotension or hypertension) as well as antihypertensive status of participants with hypertension (without medication or with medication) with chronic kidney disease incidence when the normotension was defined as individuals with normal BP (systolic BP < 130 mmHg and diastolic BP < 85 mmHg).
