# Supplementary material for: Habitual Iron Supplementation Associated with Elevated Risk of Chronic Kidney Disease in Individuals with Antihypertensive Medication
Source: Nutrients. 2024 Jul 20;16(14):2355. doi: 10.3390/nu16142355 (PMC11279651; doi:10.3390/nu16142355)
Supplement: Supplementary file 1 [file nutrients-16-02355-s001.zip › nutrients-3077187-supplementary.pdf]

Supplemental Materials

Supplemental Methods

Supplemental Table S1. Baseline characteristics of participants with hypertension in different antihypertensive status

Supplemental Table S2. Stratified analysis of the associations (HR, 95% CI) of habitual iron supplementation with risk of chronic kidney disease in participants with different hypertensive status (normotension or hypertension) and antihypertensive status (without medication or with medication)

Supplemental Table S3. Multiplicative and additive interactions between habitual iron supplementation and hypertensive status (normotension or hypertension) on chronic kidney disease risk.

Supplemental Table S4. Associations (HR, 95% CI) of habitual iron supplementation with risk of chronic kidney disease in participants with different hypertensive status (normotension or hypertension) and antihypertensive status (without medication or with medication) in the 1:4 propensity score-matched cohort*

Supplemental Table S5. Associations (HR, 95% CI) of habitual iron supplementation with risk of chronic renal failure (ICD-10 code: N18) in participants with different hypertensive status (normotension or hypertension) and antihypertensive status (without medication or with medication) **(case=17,575)**

Supplemental Table S6. Multiplicative and additive interactions between habitual iron supplementation and antihypertensive status (without medication or with medication) on chronic renal failure (ICD-10 code: N18) in participants with hypertension

Supplemental Table S7. Associations (HR, 95% CI) of habitual iron supplementation with risk of chronic kidney disease in participants with different hypertensive status

(normotension or hypertension) and antihypertensive status (without medication or with medication) after excluding people with missing covariates

Supplemental Table S8. Multiplicative and additive interactions between habitual iron supplementation and antihypertensive status (without medication or with medication) on chronic kidney disease in participants with hypertension after excluding people with missing covariates.

Supplemental Table S9. Associations (HR, 95% CI) of habitual iron supplementation with risk of chronic kidney disease in participants with different hypertensive status (normotension or hypertension) and antihypertensive (without medication or with medication) when the normotension was defined as individuals with normal BP (systolic BP<130 mmHg and diastolic BP<85 mmHg)

Supplemental Table S10. Multiplicative and additive interactions between habitual iron supplementation and antihypertensive status (without medication or with medication) on chronic kidney disease in participants with hypertension when the normotension was defined as individuals with normal BP (systolic BP<130 mmHg and diastolic BP<85 mmHg)

Supplemental Figure S1. The distribution of systolic blood pressure in participants with different hypertensive status (normotension or hypertension) and antihypertensive status (without medication or with medication)

Supplemental Figure S2. The distribution of diastolic blood pressure in participants with different hypertensive status (normotension or hypertension) and antihypertensive status (without medication or with medication)

Supplemental Figure S3. Cumulative CKD events rate varies from participants with different hypertensive status (normotension or hypertension) and antihypertensive status (without medication or with medication)

Supplemental Figure S4. Joint associations (HR, 95% CI) of habitual iron supplement use and hypertensive status (normotension or hypertension) as well as antihypertensive status of participants with hypertension (without medication or with medication) with chronic kidney disease incidence in the 1:4 propensity score-matched cohort

Supplemental Figure S5. Joint associations (HR, 95% CI) of habitual iron supplement use and hypertensive status (normotension or hypertension) as well as antihypertensive status of participants with hypertension (without medication or with medication) with chronic renal failure (ICD-10 code: N18) incidence

Supplemental Figure S6. Joint associations (HR, 95% CI) of habitual iron supplement use and hypertensive status (normotension or hypertension) as well as antihypertensive status of participants with hypertension (without medication or with medication) with chronic kidney disease incidence after excluding people with missing covariates

Supplemental Figure S7. Joint associations (HR, 95% CI) of habitual iron supplement use and hypertensive status (normotension or hypertension) as well as antihypertensive status of participants with hypertension (without medication or with medication) with chronic kidney disease incidence when the normotension was defined as individuals with normal BP (systolic BP<130 mmHg and diastolic BP<85 mmHg)

Supplemental Methods

Assessment of covariates

Information on sociodemographic characteristics were collected from central registry at recruitment and updated by the participant. The Townsend Deprivation Index (TDI), used as an indicator of socioeconomic status, based on the preceding national census output areas and calculated immediately prior to participant joining UK Biobank [62]. Other mineral supplementation refers to the use of any mineral supplements contains calcium, zinc or selenium. Similarly, vitamin supplementation refers to the use of any vitamin supplements. According to the American Heart Association recommendations on physical activity for health [40], we categorized participants into three groups based on the time spent in moderate and vigorous activity in minutes each week: inactive (no documented moderate or vigorous physical activity), insufficient (moderate activity < 150 min/week or vigorous activity < 75 min/week), and active (moderate activity >= 150 min/week or vigorous activity >= 75 min/week or equivalent combination or moderate physical activity at least 5days a week or vigorous activity once a week). The healthy diet score was defined as the following 7 groups: fruits >= 3 servings/day, vegetables >= 3 servings/day, fish >= 2 servings/day, processed meats <= 1 serving/day, unprocessed re meats <= 1.5 servings/week, whole grains >= 3 servings/day, refined grains <=1.5 servings/day [63]. For each food consumption, the participants received 1 point if meeting the criterion, and the total diet score ranged from 0 to 7. A healthy diet was defined as a diet score of 4 or more [41]. BMI was calculated as the weight (kg) divided by height squared (m^2). The drug use (aspirin, insulin, and cholesterol) was obtained through the baseline questionnaire. Prevalent diabetes was defined as a self-reported history of diabetes, the use of insulin, glycated hemoglobin A_{1c} (HbA_{1c}) >= 6.5% at baseline. The definition of hypercholesterolemia was the use of cholesterol. Anemia was defined by sex and hemoglobin concentration.

References

62. Tyrrell J, Jones SE, Beaumont R, et al. Height, body mass index, and socioeconomic status: mendelian randomisation study in UK Biobank. *BMJ*. 2016;352. doi:10.1136/bmj.i582

40. Lloyd-Jones DM, Hong Y, Labarthe D, et al. Defining and setting national goals for cardiovascular health promotion and disease reduction: the American Heart Association's strategic Impact Goal through 2020 and beyond. *Circulation*. 2010;121(4):586-613. doi:10.1161/CIRCULATIONAHA.109.192703

63. Mozaffarian D. Dietary and Policy Priorities for Cardiovascular Disease, Diabetes, and Obesity – A Comprehensive Review. *Circulation*. 2016;133(2):187-225. doi:10.1161/CIRCULATIONAHA.115.018585

41. Lourida I, Hannon E, Littlejohns TJ, et al. Association of Lifestyle and Genetic Risk With Incidence of Dementia. *JAMA*. 2019;322(5):430-437. doi:10.1001/jama.2019.9879

Supplemental Table S1. Baseline characteristics of participants with hypertension in different antihypertensive status

Variable	Total	With medication	Without medication	*p*-value
Participants, n	233 463	85 115	148 348	
CKD, n (%)	19 883 (8.5)	10 760 (12.6)	9123 (6.1)	< 0.001
Follow time, years	14.3 (2.4)	14 (2.8)	14.5 (2.1)	< 0.001
Age, years	58.6 (7.4)	60.6 (6.4)	57.5 (7.7)	< 0.001
Sex female, n (%)	113 038 (48.4)	39 898 (46.9)	73 140 (49.3)	< 0.001
TDI	-1.3 (3.1)	-1.1 (3.2)	-1.5 (3)	< 0.001
Ethnic white, n (%)	221 011 (94.7)	79 685 (93.6)	141 326 (95.3)	< 0.001
Smoking status, n (%)				< 0.001
Never	123 125 (52.7)	42 223 (49.6)	80 902 (54.5)	
Former	87 102 (37.3)	34 998 (41.1)	52 104 (35.1)	
Current	22 100 (9.5)	7454 (8.8)	14 646 (9.9)	
Alcohol drinking status, n (%)				< 0.001
Never	9969 (4.3)	4403 (5.2)	5566 (3.8)	
Former	8175 (3.5)	3672 (4.3)	4503 (3.0)	
Current	214 883 (92.0)	76 914 (90.4)	137 969 (93.0)	
Mineral supplementation, n (%)	23 204 (9.9)	7954 (9.3)	15 250 (10.3)	< 0.001
Vitamin supplementation, n (%)	72 575 (31.1)	25 167 (29.6)	47 408 (32.0)	< 0.001

Physical activity *, n (%)				< 0.001
Inactive	25 179 (10.8)	10 640 (12.5)	14 539 (9.8)	
Insufficient	37 414 (16.0)	14 976 (17.6)	22 438 (15.1)	
Active	164 896 (70.6)	56 815 (66.8)	108 081 (72.9)	
Healthy diet †, n (%)	136 523 (58.5)	49 762 (58.5)	86 761 (58.5)	< 0.001
Fruit	115 250 (50.2)	43 120 (51.5)	72 130 (49.4)	< 0.001
Vegetable	189 502 (83.0)	69 430 (83.5)	120 072 (82.7)	< 0.001
Fish	125 259 (54.2)	47 638 (56.6)	77 621 (52.8)	0.001
Processed meats	155 956 (67.0)	56 527 (66.6)	99 429 (67.2)	< 0.001
Unprocessed meats	109 426 (47.5)	38 657 (46.1)	70 769 (48.3)	0.341
Whole gains	24 706 (10.6)	9071 (10.7)	15 635 (10.6)	< 0.001
Refine gains	175 385 (75.6)	63 017 (74.6)	112 368 (76.2)	< 0.001
BMI, kg/m^2	28.4 (4.9)	29.5 (5.2)	27.8 (4.6)	< 0.001
SBP, mmHg	148.4 (15.5)	143.9 (16.9)	150.9 (14.1)	< 0.001
DBP, mmHg	86.9 (9.2)	84.1 (9.4)	88.5 (8.6)	< 0.001
Anemia, n (%)	11 843 (5.1)	5624 (6.6)	6219 (4.2)	< 0.001
HC, n (%)	56 828 (24.3)	42 098 (49.5)	14 730 (9.9)	< 0.001
Diabetes, n (%)	17 726 (7.6)	12 595 (14.8)	5131 (3.5)	< 0.001
Aspirin use, n (%)	43 258 (18.5)	30 248 (35.5)	13 010 (8.8)	< 0.001

Notes: With medication indicates participants with hypertension using antihypertensive medication. Without medication indicates participants with hypertension not using antihypertensive medication.

Data are present as mean ± SD for continuous variables and n (%) for categorical variables.

Abbreviations: CKD = chronic kidney disease; HTN = hypertension; TDI = Townsend deprivation index; BMI = body mass index; S(D)BP = systolic (diastolic) blood pressure; HC = hypercholesterolemia.

*, Physical activity was defined as inactive (no documented moderate or vigorous physical activity), insufficient (moderate activity <150 min/week and vigorous activity <75 min/week), and active (moderate activity <150 min/week and/or vigorous activity <75 min/week).

†, Healthy diet was defined as at least 4 of the following 7 food groups: fruits>=3 servings/day; vegetables>=3 servings/day; fish>=2 servings/day; processed meats<=1 serving/week; unprocessed red meats<=1.5 servings/week; whole grains>=3 servings/day; refined grains<=1.5 servings/day.

Supplemental Table S2. Stratified analysis of the associations (HR, 95% CI) of habitual iron supplementation with risk of chronic kidney disease in participants with different hypertensive status (normotension or hypertension) and antihypertensive status (without medication or with medication)

Groups	Normotension		Hypertension		Without medication		With medication	
	HR (95%CI)	*P*-value	HR (95%CI)	*P*-value	HR (95%CI)	*P*-value	HR (95%CI)	*P*-value
Age, years								
≥60	1.01 (0.83-1.25)	0.887	1.17 (1.05-1.30)	0.003	1.06 (0.90-1.26)	0.475	1.26 (1.10-1.44)	0.001
<60	1.00 (0.85-1.17)	0.978	0.94 (0.81-1.09)	0.423	0.91 (0.73-1.12)	0.367	0.98 (0.79-1.22)	0.875
Sex								
Female	1.13 (0.97-1.30)	0.110	1.07 (0.95-1.20)	0.295	1.02 (0.85-1.22)	0.855	1.13 (0.96-1.33)	0.159
Male	0.96 (0.76-1.21)	0.714	1.18 (1.04-1.33)	0.011	1.02 (0.84-1.25)	0.813	1.31 (1.11-1.53)	0.001
BMI, kg/m^2								
≥30	0.94 (0.70-1.25)	0.672	1.19 (1.04-1.36)	0.011	1.10 (0.88-1.39)	0.388	1.24 (1.05-1.46)	0.010
<30	1.09 (0.95-1.25)	0.201	1.07 (0.96-1.20)	0.227	0.99 (0.84-1.16)	0.869	1.19 (1.01-1.39)	0.037

Smoking								
Current/Former	1.03 (0.85-1.23)	0.788	1.10 (0.97-1.25)	0.121	1.06 (0.87-1.29)	0.564	1.14 (0.97-1.35)	0.123
Never	1.11 (0.94-1.31)	0.234	1.13 (1.00-1.27)	0.048	1.00 (0.84-1.21)	0.969	1.27 (1.08-1.49)	0.004
Alcohol drinking								
Current	1.07 (0.94-1.23)	0.320	1.12 (1.02-1.23)	0.020	1.05 (0.91-1.21)	0.497	1.19 (1.05-1.36)	0.008
Never/Former	1.02 (0.75-1.40)	0.882	1.11 (0.90-1.37)	0.325	0.90 (0.62-1.29)	0.560	1.24 (0.97-1.60)	0.090
Anemia								
Yes	1.00 (0.72-1.41)	0.978	1.16 (0.95-1.40)	0.139	1.11 (0.78-1.59)	0.554	1.18 (0.94-1.48)	0.148
No	1.09 (0.96-1.25)	0.187	1.09 (0.98-1.21)	0.096	1.02 (0.88-1.19)	0.752	1.17 (1.02-1.35)	0.028
Diet								
Healthy diet *	1.13 (0.97-1.31)	0.112	1.08 (0.97-1.22)	0.169	1.01 (0.85-1.20)	0.925	1.17 (1.00-1.36)	0.050
Unhealthy diet	0.92 (0.72-1.17)	0.484	1.18 (1.02-1.36)	0.030	1.02 (0.81-1.29)	0.856	1.31 (1.08-1.58)	0.005
Activity †								

Active	1.03 (0.88-1.19)	0.736	1.11 (0.99-1.23)	0.073	1.07 (0.91-1.25)	0.424	1.15 (0.99-1.34)	0.065
Inactive/Insufficient	1.14 (0.90-1.44)	0.293	1.14 (0.98-1.33)	0.094	0.95 (0.73-1.22)	0.683	1.29 (1.06-1.56)	0.010
Vitamin supplements use								
Yes	1.00 (0.87-1.15)	0.977	1.07 (0.97-1.19)	0.166	1.02 (0.88-1.18)	0.805	1.14 (1.00-1.32)	0.057
No	1.30 (1.01-1.67)	0.043	1.26 (1.06-1.50)	0.008	1.09 (0.81-1.46)	0.589	1.36 (1.10-1.67)	0.004
Diabetes								
Yes	1.86 (1.25-2.78)	0.002	1.32 (1.10-1.58)	0.003	0.95 (0.58-1.57)	0.847	1.39 (1.14-1.68)	0.001
No	1.02 (0.89-1.16)	0.818	1.05 (0.95-1.16)	0.370	1.02 (0.88-1.18)	0.819	1.10 (0.94-1.27)	0.228
Hypercholesterolemia								
Yes	1.09 (0.76-1.56)	0.653	1.32 (1.15-1.50)	<0.001	1.68 (1.26-2.23)	<0.001	1.23 (1.06-1.43)	0.006
No	1.06 (0.93-1.21)	0.355	1.00 (0.89-1.13)	0.958	0.92 (0.79-1.06)	0.251	1.18 (0.99-1.41)	0.070
Aspirin use								

Yes	0.87 (0.59-1.27)	0.468	1.40 (1.21-1.62)	<0.001	1.38 (1.02-1.87)	0.036	1.41 (1.20-1.66)	<0.001
No	1.09 (0.96-1.25)	0.180	1.00 (0.90-1.11)	0.989	0.96 (0.83-1.11)	0.590	1.06 (0.90-1.24)	0.474

HRs (95% CI) were adjusted for age (continuous), sex (male, or female), race (White, Asian, Black, mix or others), the Townsend deprivation index (continuous), alcohol drinking status (never drinking, former drinking, current drinking), smoking status (never smoker, former smoker, or current smoker), vitamin supplementation (yes, or no), other mineral supplementation (yes, or no), physical activity (inactive, insufficient, or active), healthy diet (yes, or no), BMI (continuous), anemia (yes, or no), diabetes (yes, or no), hypercholesterolemia (yes, or no), aspirin use (yes, or no). The strata variable was not included in the model when stratifying by itself.

Abbreviations: BMI = body mass index.

*, Healthy diet was defined as at least 4 of the following 7 food groups: fruits>=3 servings/day; vegetables>=3 servings/day; fish>=2 servings/day; processed meats<=1 serving/week; unprocessed red meats<=1.5 servings/week; whole grains>=3 servings/day; refined grains<=1.5 servings/day.

†, Physical activity was defined as inactive (no documented moderate or vigorous physical activity), insufficient (moderate activity <150 min/week and vigorous activity <75 min/week), and active (moderate activity <150 min/week and/or vigorous activity <75 min/week).

Supplemental Table S3. Multiplicative and additive interactions between habitual iron supplementation and hypertensive status (normotension or hypertension) on chronic kidney disease risk

	Model 1*	Model 2†	Model 3‡
**Multiplicative interaction**			
HR (95% CI)	1.09 (0.94-1.26)	1.06 (0.91-1.22)	1.05 (0.90-1.21)
P-value	0.238	0.448	0.551
**Additive interaction**			
RERI (95% CI)	0.15 (0.00-0.31)	0.10 (-0.05-0.25)	0.07 (-0.08-0.22)
P-value	0.029	0.093	0.172
AP (95% CI)	0.09 (-0.01-0.20)	0.07 (-0.04-0.18)	0.05 (-0.06-0.17)
P-value	0.039	0.106	0.182
SI (95% CI)	1.33 (0.87-2.04)	1.31 (0.75-2.28)	1.26 (0.66-2.40)
P-value	<0.001	0.005	0.021

Abbreviations: RERI = the relative excess risk due to interaction; AP = the proportion attributable to interaction; SI = the synergy index.

Model 1*: adjusted for age (continuous), sex (male, or female), race (White, Asian, Black, mix or others) and the Townsend deprivation index (continuous).

Model 2†: further adjusted for alcohol drinking status (never drinking, former drinking, current drinking), smoking status (never smoker, former smoker, or current smoker), vitamin supplementation (yes, or no), other mineral supplementation (yes, or no), physical activity (inactive, insufficient, or active), healthy diet (yes, or no), body mass index (continuous), aspirin use (yes, or no).

Model 3‡: further adjusted for anemia (yes, or no), diabetes (yes, or no), hypercholesterolemia (yes, or no).

Healthy diet was defined as at least 4 of the following 7 food groups: fruits>=3 servings/day; vegetables>=3 servings/day; fish>=2 servings/day; processed meats<=1 serving/week; unprocessed red meats<=1.5 servings/week; whole grains>=3 servings/day; refined grains<=1.5 servings/day

Supplemental Table S4. Associations (HR, 95% CI) of habitual iron supplementation with risk of chronic kidney disease in participants with different hypertensive status (normotension or hypertension) and antihypertensive status (without medication or with medication) in the 1:4 propensity score-matched cohort

Groups	Without Iron (Case/Total)	With Iron (Case/Total)	Model	
			HR (95% CI)	*P*-value
Normotension	7534/42 506 (17.7%)	275/1446 (19.0%)	1.08 (0.96-1.22)	0.211
Hypertension	17 802/84 417 (21.1%)	486/2116 (23.0%)	1.11 (1.02-1.22)	0.018
Without medication	8673/45 073 (19.2%)	228/1186 (19.2%)	1.01 (0.88-1.15)	0.915
With medication	9129/39 344 (23.2%)	258/930 (27.7%)	1.25 (1.10-1.41)	<0.001

Notes: Without iron indicates the participants not using iron supplements. With iron indicates the participants using iron supplements.
Without medication indicates participants with hypertension not using antihypertensive medication. With medication indicates participants with hypertension using antihypertensive medication.
Propensity score matching of iron supplement users with non-users was used to adjust for confounding factors including age and sex, Townsend deprivation index, race, alcohol drinking status, smoking status, physical activity, BMI, vitamin supplementation, other mineral supplementation, and healthy diet, anemia, hypercholesterolemia, aspirin use, diabetes.

Supplemental Table S5. Associations (HR, 95% CI) of habitual iron supplementation with risk of chronic renal failure (ICD-10 code: N18) in participants with different hypertensive status (normotension or hypertension) and antihypertensive status (without medication or with medication) **(case=17,575)**

Groups	Without Iron (Case/Total)	With Iron (Case/Total)	Model 1*		Model 2†		Model 3‡	
			HR (95% CI)	*P*-value	HR (95% CI)	*P*-value	HR (95% CI)	*P*-value
Normotension	3947/183 056 (2.2%)	136/7557 (1.8%)	1.00 (0.84-1.19)	0.998	1.05 (0.88-1.25)	0.587	1.03 (0.86-1.23)	0.771
Hypertension	13 094/220 856 (5.9%)	398/6216 (6.4%)	1.20 (1.09-1.33)	<0.001	1.27 (1.14-1.40)	<0.001	1.18 (1.07-1.31)	0.001
Without medication	5376/140 642 (3.8%)	148/4107 (3.6%)	1.05 (0.89-1.24)	0.536	1.12 (0.95-1.33)	0.175	1.10 (0.93-1.30)	0.287
With medication	7718/80 214 (9.6%)	250/2109 (11.9%)	1.36 (1.20-1.55)	<0.001	1.38 (1.21-1.57)	<0.001	1.25 (1.10-1.43)	0.001

Notes: Without iron indicates the participants not using iron supplements. With iron indicates the participants using iron supplements.

Without medication indicates participants with hypertension not using antihypertensive medication. With medication indicates participants with hypertension using antihypertensive medication.

Model 1*: adjusted for age (continuous), sex (male, or female), race (White, Asian, Black, mix or others) and the Townsend deprivation index (continuous).

Model 2†: further adjusted for alcohol drinking status (never drinking, former drinking, current drinking), smoking status (never smoker, former smoker, or current smoker), vitamin supplementation (yes, or no), other mineral supplementation (yes, or no), physical activity (inactive, insufficient, or active), healthy diet (yes, or no), body mass index (continuous), aspirin use (yes, or no).

Model 3‡: further adjusted for anemia (yes, or no), diabetes (yes, or no), hypercholesterolemia (yes, or no).

Healthy diet was defined as at least 4 of the following 7 food groups: fruits>=3 servings/day; vegetables>=3 servings/day; fish>=2 servings/day; processed meats<=1 serving/week; unprocessed red meats<=1.5 servings/week; whole grains>=3 servings/day; refined grains<=1.5 servings/day.

Supplemental Table S6. Multiplicative and additive interactions between habitual iron supplementation and antihypertensive status (without medication or with medication) on chronic renal failure (ICD-10 code: N18) in participants with hypertension

	Model 1*	Model 2†	Model 3‡
**Multiplicative interaction**			
HR (95% CI)	1.33 (1.08-1.63)	1.28 (1.04-1.57)	1.20 (0.98-1.48)
P-value	0.007	0.020	0.080
**Additive interaction**			
RERI (95% CI)	0.55 (0.35-0.76)	0.45 (0.26-0.64)	0.30 (0.10-0.50)
P-value	<0.001	<0.001	0.002
AP (95% CI)	0.26 (0.14-0.38)	0.24 (0.11-0.36)	0.18 (0.04-0.32)
P-value	<0.001	<0.001	0.006
SI (95% CI)	1.92 (1.14-3.22)	1.98 (1.02-3.84)	1.81 (0.82-3.96)
P-value	<0.001	<0.001	<0.001

Abbreviations: RERI = the relative excess risk due to interaction; AP = the proportion attributable to interaction; SI = the synergy index.

Model 1*: adjusted for age (continuous), sex (male, or female), race (White, Asian, Black, mix or others) and the Townsend deprivation index (continuous).

Model 2†: further adjusted for alcohol drinking status (never drinking, former drinking, current drinking), smoking status (never smoker, former smoker, or current smoker), vitamin supplementation (yes, or no), other mineral supplementation (yes, or no), physical activity (inactive, insufficient, or active), healthy diet (yes, or no), body mass index (continuous), aspirin use (yes, or no).

Model 3‡: further adjusted for anemia (yes, or no), diabetes (yes, or no), hypercholesterolemia (yes, or no).

Healthy diet was defined as at least 4 of the following 7 food groups: fruits>=3 servings/day; vegetables>=3 servings/day; fish>=2 servings/day; processed meats<=1 serving/week; unprocessed red meats<=1.5 servings/week; whole grains>=3 servings/day; refined grains<=1.5 servings/day.

Supplemental Table S7. Associations (HR, 95% CI) of habitual iron supplementation with risk of chronic kidney disease in participants with different hypertensive status (normotension or hypertension) and antihypertensive status (without medication or with medication) after excluding people with missing covariates

Groups	Without Iron (Case/Total)	With Iron (Case/Total)	Model 1*		Model 2†		Model 3‡	
			HR (95% CI)	*P*-value	HR (95% CI)	*P*-value	HR (95% CI)	*P*-value
Normotension	6370/159 535 (4.0%)	228/6465 (3.5%)	1.03 (0.92-1.16)	0.594	1.04 (0.91-1.19)	0.535	1.04 (0.91-1.20)	0.538
Hypertension	15 628/189 275 (8.3%)	431/5139 (8.4%)	1.14 (1.05-1.24)	0.003	1.16 (1.06-1.28)	0.001	1.11 (1.00-1.22)	0.045
Without medication	7124/120 405 (5.9%)	187/3450 (5.4%)	1.01 (0.89-1.15)	0.885	1.03 (0.89-1.19)	0.693	1.03 (0.89-1.20)	0.685
With medication	8504/68 870 (12.3%)	244/1689 (14.4%)	1.29 (1.16-1.44)	<0.001	1.31 (1.15-1.48)	<0.001	1.19 (1.04-1.35)	0.011

Notes: Without iron indicates the participants not using iron supplements. With iron indicates the participants using iron supplements.

Without medication indicates participants with hypertension not using antihypertensive medication. With medication indicates participants with hypertension using antihypertensive medication.

Model 1*: adjusted for age (continuous), sex (male, or female), race (White, Asian, Black, mix or others) and the Townsend deprivation index (continuous).

Model 2†: further adjusted for alcohol drinking status (never drinking, former drinking, current drinking), smoking status (never smoker, former smoker, or current smoker), vitamin supplementation (yes, or no), other mineral supplementation (yes, or no), physical activity (inactive, insufficient, or active), healthy diet (yes, or no), body mass index (continuous), aspirin use (yes, or no).

Model 3‡: further adjusted for anemia (yes, or no), diabetes (yes, or no), hypercholesterolemia (yes, or no).

Healthy diet was defined as at least 4 of the following 7 food groups: fruits>=3 servings/day; vegetables>=3 servings/day; fish>=2 servings/day; processed meats<=1 serving/week; unprocessed red meats<=1.5 servings/week; whole grains>=3 servings/day; refined grains<=1.5 servings/day.

Supplemental Table S8. Multiplicative and additive interactions between habitual iron supplementation and antihypertensive status (without medication or with medication) on chronic kidney disease in participants with hypertension after excluding people with missing covariates

	**Model 1***	**Model 2†**	**Model 3‡**
**Multiplicative interaction**			
HR (95% CI)	1.3 (1.07-1.58)	1.26 (1.04-1.53)	1.19 (0.98-1.45)
P-value	0.008	0.018	0.074
**Additive interaction**			
RERI (95% CI)	0.41 (0.22-0.61)	0.34 (0.16-0.53)	0.24 (0.05-0.43)
P-value	<0.001	<0.001	0.007
AP (95% CI)	0.23 (0.10-0.36)	0.21 (0.08-0.35)	0.16 (0.02-0.31)
P-value	<0.001	0.001	0.014
SI (95% CI)	2.07 (0.98-4.35)	2.27 (0.81-6.38)	2.04 (0.63-6.62)
P-value	<0.001	<0.001	0.001

Abbreviations: RERI = the relative excess risk due to interaction; AP = the proportion attributable to interaction; SI = the synergy index.

Model 1*: adjusted for age (continuous), sex (male, or female), race (White, Asian, Black, mix or others) and the Townsend deprivation index (continuous).

Model 2†: further adjusted for alcohol drinking status (never drinking, former drinking, current drinking), smoking status (never smoker, former smoker, or current smoker), vitamin supplementation (yes, or no), other mineral supplementation (yes, or no), physical activity (inactive, insufficient, or active), healthy diet (yes, or no), body mass index (continuous), aspirin use (yes, or no).

Model 3‡: further adjusted for anemia (yes, or no), diabetes (yes, or no), hypercholesterolemia (yes, or no).

Healthy diet was defined as at least 4 of the following 7 food groups: fruits>=3 servings/day; vegetables>=3 servings/day; fish>=2 servings/day; processed meats<=1 serving/week; unprocessed red meats<=1.5 servings/week; whole grains>=3 servings/day; refined grains<=1.5 servings/day.

Supplemental Table S9. Associations (HR, 95% CI) of habitual iron supplementation with risk of chronic kidney disease in participants with different hypertensive status (normotension or hypertension) and antihypertensive (without medication or with medication) when the normotension was defined as individuals with normal BP (systolic BP<130 mmHg and diastolic BP<85 mmHg)

Groups	Without Iron (Case/Total)	With Iron (Case/Total)	Model 1*		Model 2†		Model 3‡	
			HR (95% CI)	*P*-value	HR (95% CI)	*P*-value	HR (95% CI)	*P*-value
Normotension	4528/120 199 (3.8%)	190/5523 (3.4%)	1.06 (0.91-1.22)	0.470	1.08 (0.93-1.26)	0.290	1.07 (0.92-1.25)	0.365
Hypertension	22 458/293 658 (7.6%)	653/8559 (7.6%)	1.11 (1.03-1.20)	0.008	1.15 (1.06-1.25)	0.001	1.11 (1.02-1.20)	0.014
Without medication	12 021/210 725 (5.7%)	330/6377 (5.2%)	1.01 (0.90-1.12)	0.913	1.04 (0.93-1.17)	0.472	1.03 (0.92-1.15)	0.624
With medication	10 437/82 933 (12.6%)	323/2182 (14.8%)	1.29 (1.15-1.44)	<0.001	1.30 (1.16-1.45)	<0.001	1.21 (1.08-1.35)	0.001

Notes: Without iron indicates the participants not using iron supplements. With iron indicates the participants using iron supplements.
Without medication indicates participants with hypertension not using antihypertensive medication. With medication indicates participants with hypertension using antihypertensive medication. BP = blood pressure.

Model 1*: adjusted for age (continuous), sex (male, or female), race (White, Asian, Black, mix or others) and the Townsend deprivation index (continuous).

Model 2†: further adjusted for alcohol drinking status (never drinking, former drinking, current drinking), smoking status (never smoker, former smoker, or current smoker), vitamin supplementation (yes, or no), other mineral supplementation (yes, or no), physical activity (inactive, insufficient, or active), healthy diet (yes, or no), body mass index (continuous), aspirin use (yes, or no).

Model 3‡: further adjusted for anemia (yes, or no), diabetes (yes, or no), hypercholesterolemia (yes, or no).

Healthy diet was defined as at least 4 of the following 7 food groups: fruits>=3 servings/day; vegetables>=3 servings/day; fish>=2 servings/day; processed meats<=1 serving/week; unprocessed red meats<=1.5 servings/week; whole grains>=3 servings/day; refined grains<=1.5 servings/day.

Supplemental Table S10. Multiplicative and additive interactions between habitual iron supplementation and antihypertensive status (without medication or with medication) on chronic kidney disease in participants with hypertension when the normotension was defined as individuals with normal BP (systolic BP<130 mmHg and diastolic BP<85 mmHg)

	**Model 1***	**Model 2†**	**Model 3‡**
**Multiplicative interaction**			
HR (95% CI)	1.30 (1.11-1.52)	1.26 (1.08-1.47)	1.20 (1.03-1.41)
P-value	0.001	0.004	0.020
**Additive interaction**			
RERI (95% CI)	0.43 (0.26-0.59)	0.34 (0.18-0.49)	0.25 (0.10-0.41)
P-value	<0.001	<0.001	0.001
AP (95% CI)	0.23 (0.13-0.34)	0.21 (0.10-0.32)	0.17 (0.05-0.29)
P-value	<0.001	<0.001	0.002
SI (95% CI)	2.04 (1.16-3.58)	2.23 (1.00-4.98)	2.15 (0.80-5.79)
P-value	<0.001	<0.001	<0.001

Abbreviations:BP = blood pressure; RERI = the relative excess risk due to interaction; AP = the proportion attributable to interaction; SI = the synergy index.

Model 1*: adjusted for age (continuous), sex (male, or female), race (White, Asian, Black, mix or others) and the Townsend deprivation index (continuous).

Model 2†: further adjusted for alcohol drinking status (never drinking, former drinking, current drinking), smoking status (never smoker, former smoker, or current smoker), vitamin supplementation (yes, or no), other mineral supplementation (yes, or no), physical activity (inactive, insufficient, or active), healthy diet (yes, or no), body mass index (continuous), aspirin use (yes, or no).

Model 3‡: further adjusted for anemia (yes, or no), diabetes (yes, or no), hypercholesterolemia (yes, or no).

Healthy diet was defined as at least 4 of the following 7 food groups: fruits>=3 servings/day; vegetables>=3 servings/day; fish>=2 servings/day; processed meats<=1 serving/week; unprocessed red meats<=1.5 servings/week; whole grains>=3 servings/day; refined grains<=1.5 servings/day.

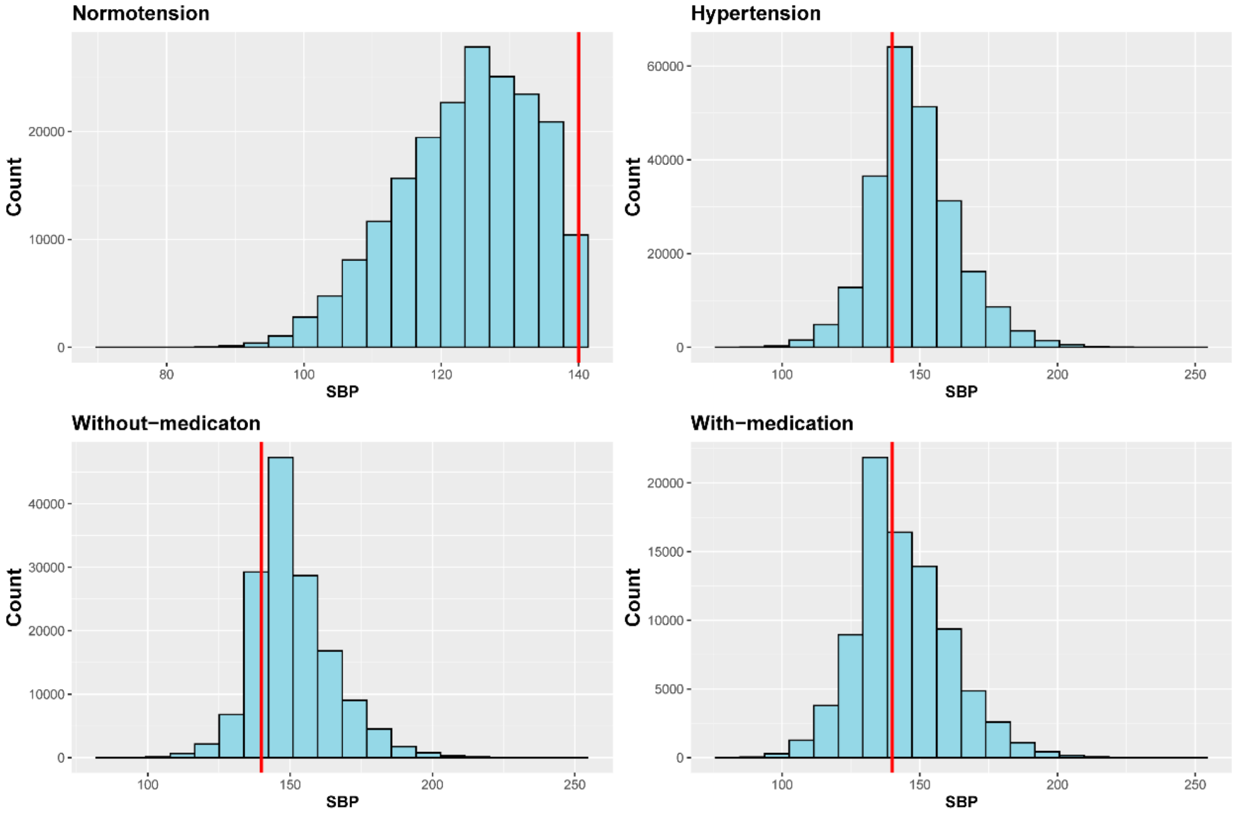

Supplemental Figure S1. The distribution of systolic blood pressure in participants with different hypertensive status (normotension or hypertension) and antihypertensive status (without medication or with medication)

Notes: Without medication indicates participants with hypertension not using antihypertensive medication. With medication indicates participants with hypertension using antihypertensive medication.

Abbreviations: SBP = systolic blood pressure. The red vertical line indicates that the SBP is 140mmHg.

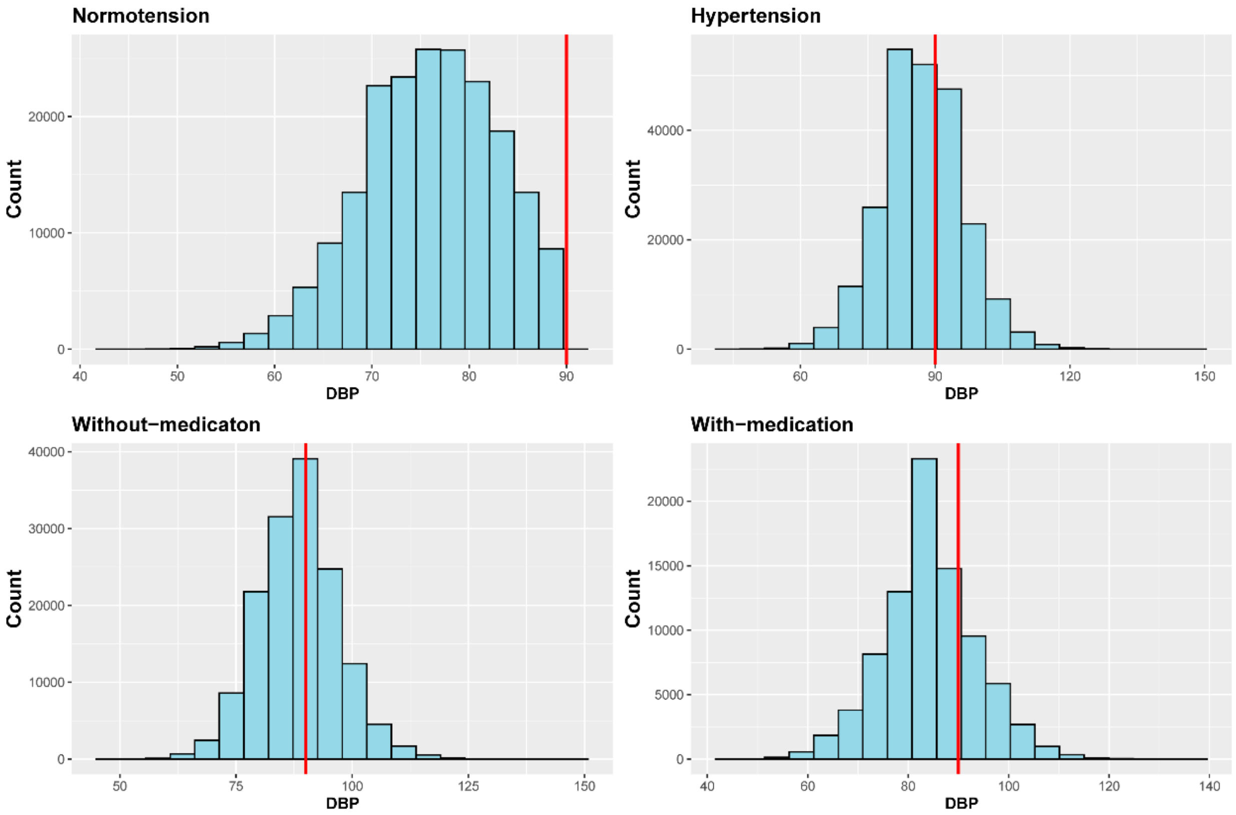

Supplemental Figure S2. The distribution of diastolic blood pressure in participants with different hypertensive status (normotension or hypertension) and antihypertensive status (without medication or with medication)

Notes: Without medication indicates participants with hypertension not using antihypertensive medication. With medication indicates participants with hypertension using antihypertensive medication.

Abbreviations: DBP = diastolic blood pressure. The red vertical line indicates that the DBP is 90mmHg.

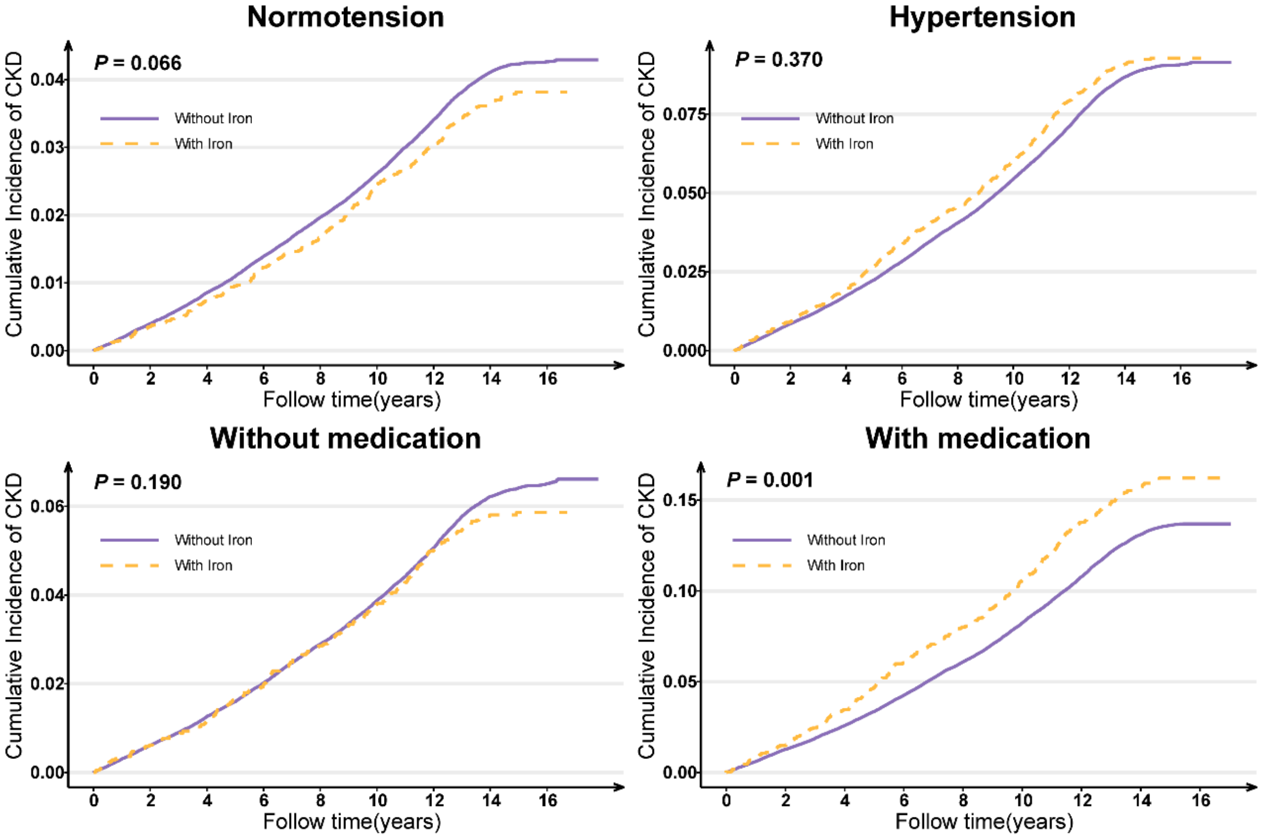

Supplemental Figure S3. Cumulative CKD events rate varies from participants with different hypertensive status (normotension or hypertension) and antihypertensive status (without medication or with medication)

Notes: Without iron indicates the participants not using iron supplements. With iron indicates the participants using iron supplements. Without medication indicates participants with hypertension not using antihypertensive medication. With medication indicates participants with hypertension using antihypertensive medication.

Abbreviations: CKD = chronic kidney disease.

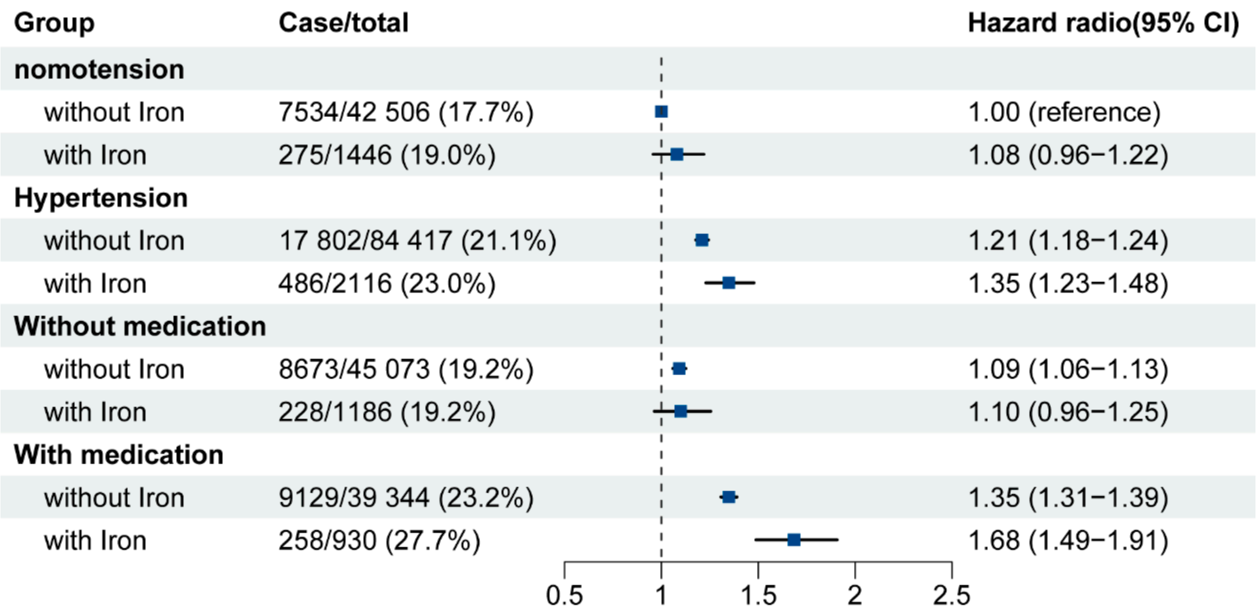

Supplemental Figure S4. Joint associations (HR, 95% CI) of habitual iron supplement use and hypertensive status (normotension or hypertension) as well as antihypertensive status of participants with hypertension (without medication or with medication) with chronic kidney disease incidence in the 1:4 propensity score-matched cohort

Notes: Without iron indicates the participants not using iron supplements. With iron indicates the participants using iron supplements. Without medication indicates participants with hypertension not using antihypertensive medication. With medication indicates participants with hypertension using antihypertensive medication.

HRs (95% CI) were adjusted for age (continuous), sex (male, or female), race (White, Asian, Black, mix or others), the Townsend deprivation index (continuous), alcohol drinking status (never drinking, former drinking, current drinking), smoking status (never smoker, former smoker, or current smoker), vitamin supplementation (yes, or no), other mineral supplementation (yes, or no), physical activity (inactive, insufficient, or active), healthy diet (yes, or no), BMI (continuous), anemia (yes, or no), diabetes (yes, or no), hypercholesterolemia (yes, or no), aspirin use (yes, or no).
Healthy diet was defined as at least 4 of the following 7 food groups: fruits>=3 servings/day; vegetables>=3 servings/day; fish>=2 servings/day; processed meats<=1 serving/week; unprocessed red meats<=1.5 servings/week; whole grains>=3 servings/day; refined grains<=1.5 servings/day.

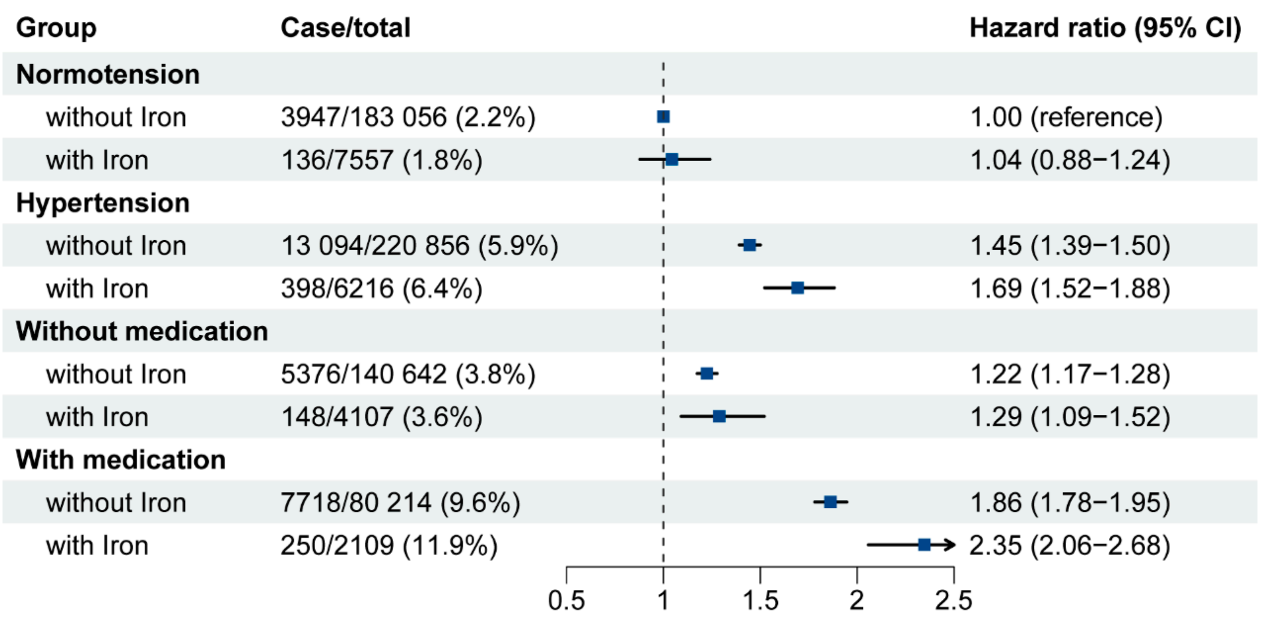

Supplemental Figure S5. Joint associations (HR, 95% CI) of habitual iron supplement use and hypertensive status (normotension or hypertension) as well as antihypertensive status of participants with hypertension (without medication or with medication) with chronic renal failure (ICD-10 code: N18) incidence

Notes: Without iron indicates the participants not using iron supplements. With iron indicates the participants using iron supplements. Without medication indicates participants with hypertension not using antihypertensive medication. With medication indicates participants with hypertension using antihypertensive medication.

HRs (95% CI) were adjusted for age (continuous), sex (male, or female), race (White, Asian, Black, mix or others), the Townsend deprivation index (continuous), alcohol drinking status (never drinking, former drinking, current drinking), smoking status (never smoker, former smoker, or current smoker), vitamin supplementation (yes, or no), other mineral supplementation (yes, or no), physical activity (inactive, insufficient, or active), healthy diet (yes, or no), BMI (continuous), anemia (yes, or no), diabetes (yes, or no), hypercholesterolemia (yes, or no), aspirin use (yes, or no).

Healthy diet was defined as at least 4 of the following 7 food groups: fruits>=3 servings/day; vegetables>=3 servings/day; fish>=2 servings/day; processed meats<=1 serving/week; unprocessed red meats<=1.5 servings/week; whole grains>=3 servings/day; refined grains<=1.5 servings/day.

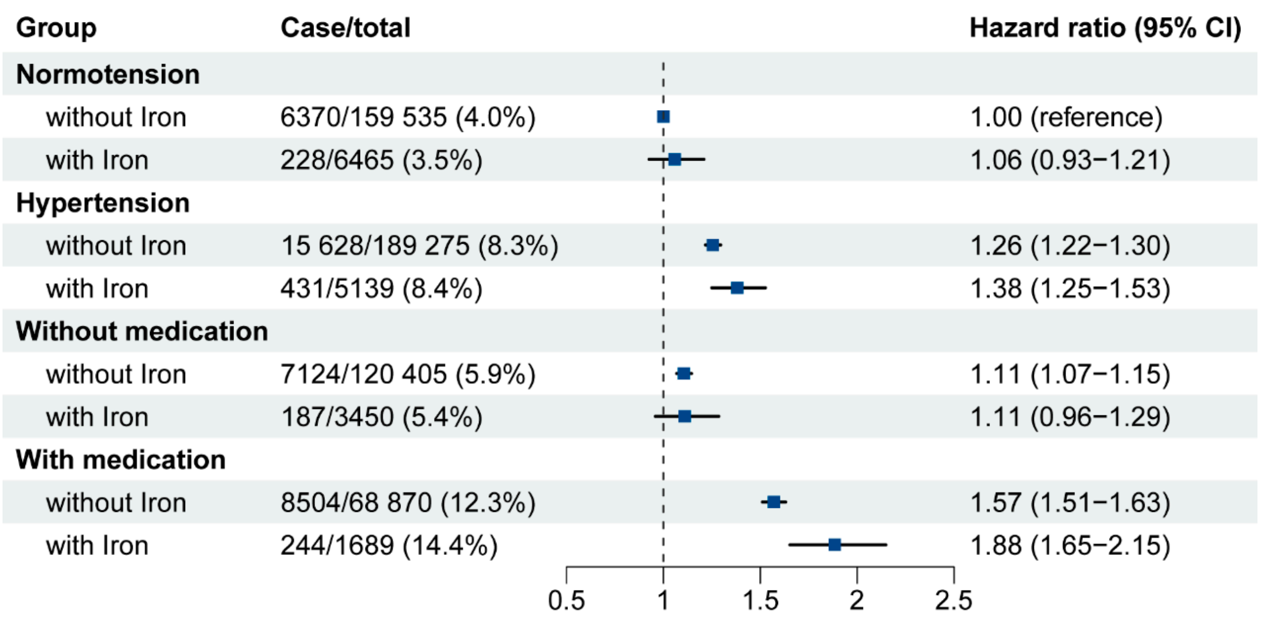

Supplemental Figure S6. Joint associations (HR, 95% CI) of habitual iron supplement use and hypertensive status (normotension or hypertension) as well as antihypertensive status of participants with hypertension (without medication or with medication) with chronic kidney disease incidence after excluding people with missing covariates

Notes: Without iron indicates the participants not using iron supplements. With iron indicates the participants using iron supplements. Without medication indicates participants with hypertension not using antihypertensive medication. With medication indicates participants with hypertension using antihypertensive medication.

HRs (95% CI) were adjusted for age (continuous), sex (male, or female), race (White, Asian, Black, mix or others), the Townsend deprivation index (continuous), alcohol drinking status (never drinking, former drinking, current drinking), smoking status (never smoker, former smoker, or current smoker), vitamin supplementation (yes, or no), other mineral supplementation (yes, or no), physical activity (inactive, insufficient, or active), healthy diet (yes, or no), BMI (continuous), anemia (yes, or no), diabetes (yes, or no), hypercholesterolemia (yes, or no), aspirin use (yes, or no).

Healthy diet was defined as at least 4 of the following 7 food groups: fruits>=3 servings/day; vegetables>=3 servings/day; fish>=2 servings/day; processed meats<=1 serving/week; unprocessed red meats<=1.5 servings/week; whole grains>=3 servings/day; refined grains<=1.5 servings/day.

Group	Case/total	Hazard ratio (95% CI)
Normotension		
without Iron	4528/120 199 (3.8%)	1.00 (reference)
with Iron	190/5523 (3.4%)	1.09 (0.95−1.27)
Hypertension		
without Iron	22 458/293 658 (7.6%)	1.21 (1.17−1.25)
with Iron	653/8559 (7.6%)	1.33 (1.22−1.44)
Without medication		
without Iron	12 021/210 725 (5.7%)	1.10 (1.06−1.14)
with Iron	330/6377 (5.2%)	1.11 (0.99−1.24)
With medication		
without Iron	10 437/82 933 (12.6%)	1.56 (1.50−1.63)
with Iron	323/2182 (14.8%)	1.90 (1.69−2.14)

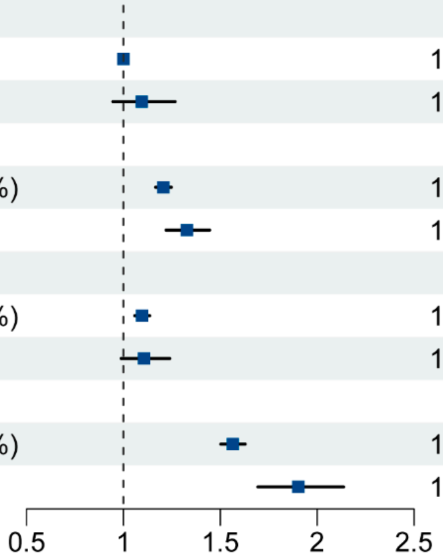

Supplemental Figure S7. Joint associations (HR, 95% CI) of habitual iron supplement use and hypertensive status (normotension or hypertension) as well as antihypertensive status of participants with hypertension (without medication or with medication) with chronic kidney disease incidence when the normotension was defined as individuals with normal BP (systolic BP<130 mmHg and diastolic BP<85 mmHg)

Notes: Without iron indicates the participants not using iron supplements. With iron indicates the participants using iron supplements. Without medication indicates participants with hypertension not using antihypertensive medication. With medication indicates participants with hypertension using antihypertensive medication. BP = blood pressure.

HRs (95% CI) were adjusted for age (continuous), sex (male, or female), race (White, Asian, Black, mix or others), the Townsend deprivation index (continuous), alcohol drinking status (never drinking, former drinking, current drinking), smoking status (never smoker, former smoker, or current smoker), vitamin supplementation (yes, or no), other mineral supplementation (yes, or no), physical activity (inactive, insufficient, or active), healthy diet (yes, or no), BMI (continuous), anemia (yes, or no), diabetes (yes, or no), hypercholesterolemia (yes, or no), aspirin use (yes, or no).
Healthy diet was defined as at least 4 of the following 7 food groups: fruits>=3 servings/day; vegetables>=3 servings/day; fish>=2 servings/day; processed meats<=1 serving/week; unprocessed red meats<=1.5 servings/week; whole grains>=3 servings/day; refined grains<=1.5 servings/day.
